# Hospitals Safety from Disasters in I.R.Iran: The Results from Assessment of 224 Hospitals

**DOI:** 10.1371/currents.dis.8297b528bd45975bc6291804747ee5db

**Published:** 2014-02-28

**Authors:** Ali Ardalan, Maryam Kandi, Mohammad Taghi Talebian, Hamidreza Khankeh, Gholamreza Masoumi, Reza Mohammadi, Samaneh Maleknia, Jafar Miadfar, Atieh Mobini, Sara Mehranamin

**Affiliations:** Department of Disaster & Emergency Health, Iran's National Institute of Health Research; Department of Disaster Public Health, School of Public Health, Tehran University of Medical Sciences, Tehran, Iran; Harvard Humanitarian Initiative, Harvard University; Department of Disaster & Emergency Health, National Institute of Health Research, Tehran University of Medical Sciences, Tehran, Iran; Disaster & Emergency Management Center, Ministry of Health & Medical Education, Tehran, Iran; Department of Disaster & Emergency Health, University of Social Welfare and Rehabilitation, Tehran, Iran; Department of Clinical Science and Education, Karolinska Institute, Stockholm, Sweden; Disaster and Emergency Management Center, Ministry of Health & Medical Education, Iran; Disaster & Emergency Management Center, Ministry of Health & Medical Education, Tehran, Iran; Disaster & Emergency Management Center, Ministry of Health & Medical Education, Tehran, Iran; Disaster & Emergency Management Center, Ministry of Health & Medical Education, Tehran, Iran; Disaster & Emergency Management Center, Ministry of Health & Medical Education, Tehran, Iran; Disaster & Emergency Management Center, Ministry of Health & Medical Education, Tehran, Iran

## Abstract

Background and objective: Iran’s hospitals have been considerably affected by disasters during last decade. To address this, health system of I.R.Iran has taken an initiative to assess disaster safety of the hospitals using an adopted version of Hospital Safety Index (HSI). This article presents the results of disaster safety assessment in 224 Iran’s hospitals. Methods: A self-assessment approach was applied to assess the disaster safety in 145 items categorized in 3 components including structural, non-structural and functional capacity. For each item, safety level was categorized to 3 levels: not safe (0), average safe (1) and high safe (2). A raw score was tallied for each safety component and its elements by a simple sum of all the corresponding scores. All scores were normalized on a 100 point scale. Hospitals were classified to three safety classes according to their normalized total score: low (≤34.0), average (34.01-66.0) and high (>66.0). Results: The average score of all safety components were 32.4 out of 100 (± 12.7 SD). 122 hospitals (54.5%) were classified as low safe and 102 hospitals (45.5%) were classified as average safe. No hospital was placed in the high safe category. Average safety scores out of 100 were 27.3 (±14.2 SD) for functional capacity, 36.0 (±13.9 SD) for non-structural component and 36.0 (±19.0 SD) for structural component. Neither the safety classes nor the scores of safety components were significantly associated with types of hospitals in terms of affiliation, function and size (P>0.05). Conclusions: To enhance the hospitals safety for disaster in Iran, we recommend: 1) establishment of a national committee for hospital safety in disasters; 2) supervision on implementation of the safety standards in construction of new hospitals; 3) enhancement of functional readiness and safety of non-structural components while structural retrofitting of the existing hospitals is being taken into consideration, whenever is cost-effective; 4) considering the disaster safety status as the criteria for licensing and accreditation of the hospitals. Key words: Hospital, safety, disaster, emergency, Iran Correspondence to: Ali Ardalan MD, PhD. Tehran University of Medical Sciences, Harvard Humanitarian Initiative, Email: aardalan@tums.ac.ir, ardalan@hsph.harvard.edu

## Introduction

Iran (the Islamic Republic) is a Middle East country covering an area of 1,648 million sq km with a population of about 75 million. It is exposed to a variety of natural and man-made disasters that have caused considerable damages to the population and infrastructures. According to the Global Assessment Report on Disaster Reduction 1, the Iran’s risk class for natural hazards is 8 out of 10. Over last four decades, these hazards have caused more than 109,000 deaths and 150,000 injuries in Iran 2.

Adverse impacts of disasters on the Iranian hospitals have been enormous. For example, in the Bam earthquake (2003), almost all public and private hospitals were totally collapsed. The Zarand earthquake (2005) led to non-functionality of the district hospital for about six hours due to non-structural damages and absence of staff. In the East Azarbaijan earthquake (2012), the district hospitals were almost collapsed including one hospital that had been opened only one year before the earthquake. A fire accident in the Arg mosque of Tehran (2006) that led to more than 100 burn injuries challenged the Tehran’s hospitals for their surge capacity and managing the burned cases. A similar problem was observed following a bomb explosion in Shiraz (2009) that left 202 injuries.

Hospital safety from disasters is a challenge in both developing and developed countries 3 ^-^ 6. During disasters, the hospitals must be able to continue their functions in a safe environment and save the lives of injured victims 7. Hospitals are potentially vulnerable to disasters because of their complexity in terms of structural, non-structural and functional components; high level of occupancy and expensive equipment 8.

To facilitate the process of hospital safety assessment for disasters, the World Health Organization (WHO) has developed the Hospital Safety Index (HSI) 7. According to WHO, the HSI is a rapid, reliable and low-cost diagnostic tool that addresses the structural safety, non-structural safety and functional capacity of a hospital in 145 areas. While the HSI does not replace detailed vulnerability studies, it provides the decision makers with an overall idea of the hospitals’ ability to respond to major emergencies and disasters. In other word, an assessment using the HSI is the first step toward prioritizing a country’s investments in hospital safety. This helps the decision makers to prioritize the resource allocation 7.

In 2011, a multidisciplinary group of experts, including doctors, nurses, disaster managers and engineers gathered in Tehran University of Medical Sciences (TUMS) to help the Iran’s Ministry of Health and Medical Education (MOH&ME) for developing an adopted version of HSI that works in Iran’s situation. Accordingly, the HSI was translated to Farsi and tested in pilot hospitals. The content validity of the tool was assessed based on opinions of the subject experts and the face validity was assessed using views of the hospital personnel. The adopted tool was called Farsi Hospital Safety Index (FHSI). To be comparable with the assessments in other countries, in FHSI, we kept the structure of the tool and number of items similar to the original HSI version. However, the modifications were made in the section of guides to evaluators whenever appropriate.

In 2012, all 919 Iran’s hospitals were mandated by MOH&ME to assess their safety status using FHSI and report back to MOH&ME. By September 2013, 224 hospitals (24.4%) completed their assessment. This article presents the results of the safety assessment for disasters in 224 Iran’s hospitals using FHSI. The findings have applications for prioritizing the resource allocation and interventions by decision makers.

## Methods

The survey was conducted from August 2012 through August 2013 through a self-assessment approach. Accordingly, in each hospital the hospital disaster committee (HDC) was responsible for the assessment coordination, data collection and data entry and reporting to MOH&ME. The assessment team included three to five members of the HDC including doctors, nurses and technicians or engineers from hospital maintenance office. During the survey period, two officers at the MOH&ME were available during working hours to answer queries from the data collection team. Data were entered into an Excel spreadsheet and uploaded to the portal system of MOH&ME.

Safety assessment was conducted in three components including structural (13 items in 2 elements), non-structural (71 items in 9 elements) and functional capacity (61 items in 5 elements). In addition to safety components, the assessors were also instructed to fill in the forms related to general information of the hospital and hazards identification. Table 1 presents the elements of each safety component along with number of items for each element.

**Table 1 – Components of hospital safety in Farsi Hospital Safety Index:** 

**Safety component **	**Safety element **	**Number of items**
Structural	Previous events affecting the safety of hospital buildings	3
	Safety of structural systems and materials used in building	10
Non-structural	Electrical system	8
	Telecommunications system	7
	Water supply	5
	Fuel storage	4
	Medical gases	7
	Heating, ventilation, and air-conditioning (HVAC) systems in critical areas	7
	Office and storeroom furnishings and equipment (fixed and movable) including computers, printers, etc.	3
	Medical and laboratory equipment and supplies used for diagnosis and treatment	12
	Architectural elements	18
Functional capacity	Organization of Hospital Disaster Committee and Emergency Operations Center	11
	Operational plan for internal or external disasters	24
	Contingency plans for medical treatment in disasters	8
	Plans for the operation, preventive maintenance, and restoration of critical services	8
	Availability of medicines, supplies, instruments, and other equipment for use in emergency	10
Total		145

For each item, the safety status was categorized to three levels: not safe, average safe and high safe. The scores of 0, 1 and 2 were assigned to them, respectively. In each safety component (functional, non-structural and structural) and corresponding elements, equal weight was given to each item and a raw score was tallied by a simple sum of all the items scores. To calculate the total safety score of hospitals, equal weight was given to each component. All scores were normalized on a 100 point scale. Finally, hospitals were classified to three safety classes according to their corresponding normalized total score as follow: low (≤34.0), average (34.01-66.0) and high (>66.0).

Using the normalized safety scores, the hospitals were compared according to their affiliation (Ministry of Health, private, Social Welfare Organization, Military and charity), function (general *vs.* specialized) and size (≤100 *vs.* >100 beds). One-way ANOVA, independent t-test and chi-square were the statistical tests where appropriate and SPSS 11.0 was used for statistical analysis. P<0.05 was considered as statistically significant.

## Results

From August 2012 to August 2013, 224 Iranian hospitals reported their safety status to MOH&ME using FHSI (24.4% response rate). Table 2 presents the characteristics of the assessed hospitals.

**Table 2 – Characteristics of 224 Iran’s hospitals that assessed their safety status for disasters, 2012-2013:** *MOH&ME: Ministry of Health & Medical Education †SWO: Social Welfare Organization

	**n**	**%**
**Hospital affiliation **		
MOH&ME*	171	76.3
Private	25	11.2
SWO†	20	8.9
Military	5	2.2
Charity	3	1.3
**Hospital function**		
General	180	80.4
Specialized	44	19.6
**Hospital size**		
≤100 beds	181	80.8
>100 beds	43	19.2

The average score of all safety components were 32.4 out of 100 (± 12.7 SD). One hundred and twenty two hospitals (54.5%) were classified as low safe and 102 hospitals (45.5%) were classified as average safe (table 3). No hospital was placed in the high safe category.

**Table 3 – Disaster safety class in 224 Iran’s hospitals, 2012-2013:** *MOH&ME: Ministry of Health & Medical Education †SWO: Social Welfare Organization

	**Low safety**	**Moderate safety**	**High safety**	**Total**	**P value**
	**n (%)**	**n (%)**	**n (%)**	**n (%)**	
**Hospital affiliation **					0.84
MOH&ME*	90 (52.6)	81 (47.4)	0	171 (100)	
Private	14 (56.0)	11 (44.0)	0	25 (100)	
SWO†	13 (65.0)	7 (35.0)		20 (100)	
Military	3 (60.0)	2 (40.0)	0	5 (100)	
Charity	2 (66.7)	1 (33.3)	0	3 (100)	
**Hospital function**					0.30
General	95 (52.8)	85 (47.2)	0	180 (100)	
Specialized	27 (61.4)	17 (38.6)	0	44 (100)	
**Hospital size**					0.24
≤100 beds	102 (56.4)	79 (43.6)	0	181 (100)	
>100 beds	20 (46.5)	23 (53.5)	0	43 (100)	
**All hospitals **	122 (54.5)	102 (45.5)	0	224 (100)	

Regarding the various elements of functional capacity, as presented in table 4, the average safety score ranged from 20.2 to 38.0 for the “contingency plans for medical treatment in disasters” and “availability of medicines, supplies, instruments, and other equipment for use in emergency”, respectively. The average safety score was 27.3 out of 100 (±14.2 SD) for all elements of functional capacity. Figure 1-A visualizes the safety scores of hospitals in terms of various elements of functional capacity.

**Table 4 – Average safety score of functional capacity for disasters in 224 Iran’s hospitals, 2012-2013:** *MOH&ME: Ministry of Health & Medical Education; †SWO: Social Welfare Organization; F1: Organization of Hospital Disaster Committee and Emergency Operations Center; F2: Operational plan for internal or external disasters; F3: Contingency plans for medical treatment in disasters; F4: Plans for the operation, preventive maintenance, and restoration of critical services; F5: Availability of medicines, supplies, instruments, and other equipment for use in emergency

	**F1**	**F2**	**F3**	**F4**	**F5**	**All elements**
**Hospital affiliation **						
MOH&ME*	35.0	25.6	20.3	35.0	38.1	27.5
Private	30.9	25.4	20.3	38.4	42.6	28.7
SWO†	28.1	21.8	17.0	29.4	30.7	24.1
Military	42.7	35.5	27.4	20.3	40.6	33.3
Charity	34.2	15.7	19.6	37.5	38.2	19.8
*P value*	*0.36*	*0.33*	*0.74*	*0.24*	*0.23*	*0.75*
**Hospital function**						
General	34.3	25.7	20.1	34.0	38.1	27.2
Specialized	33.1	24.1	20.3	36.9	37.9	27.7
*P value*	*0.72*	*0.53*	*0.94*	*0.37*	*0.95*	*0.86*
**Hospital size**						
≤100 beds	32.77	24.6	19.1	33.4	37.3	26.5
>100 beds	39.6	28.4	24.9	39.1	41.2	30.6
*P value*	*0.03*	*0.14*	*0.02*	*0.06*	*0.18*	*0.09*
**All hospitals **	34.1	25.4	20.2	34.6	38.0	27.3

**Disaster safety score in terms of A) functional capacity, B) non-structural components and C) structural components in 224 Iran’s hospitals, 2012-2013:**
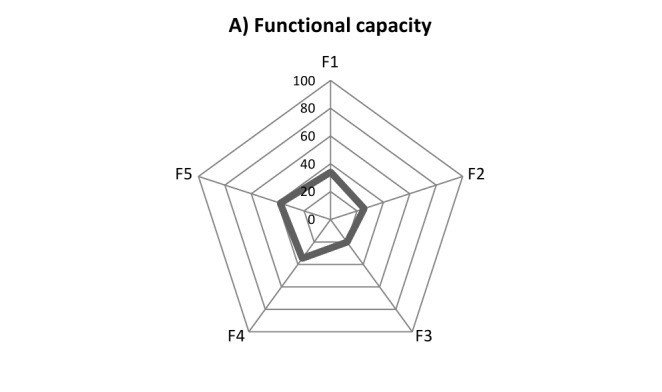
F1: Organization of Hospital Disaster Committee and Emergency Operations Center F2: Operational plan for internal or external disasters F3: Contingency plans for medical treatment in disasters F4: Plans for the operation, preventive maintenance, and restoration of critical services F5: Availability of medicines, supplies, instruments, and other equipment for use in emergency

**Figure:**
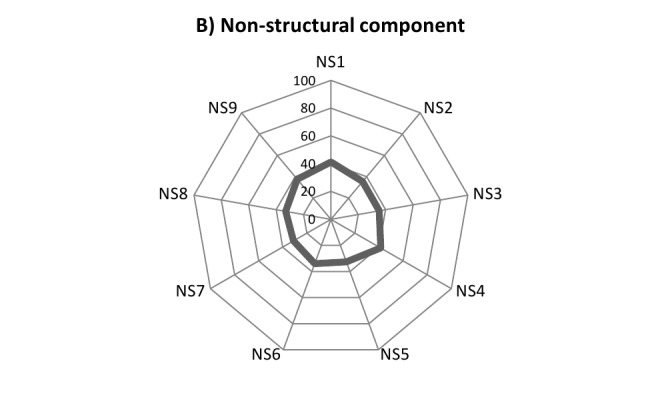
NS1: Electrical system NS2: Telecommunications system NS3: Water supply NS4: Fuel storage NS5: Medical gases NS6: Heating, ventilation, and air-conditioning (HVAC) systems in critical areas NS7: Office and storeroom furnishings and equipment (fixed and movable) NS8: Medical and laboratory equipment and supplies used for diagnosis and treatment NS9: Architectural elements

**Figure:**
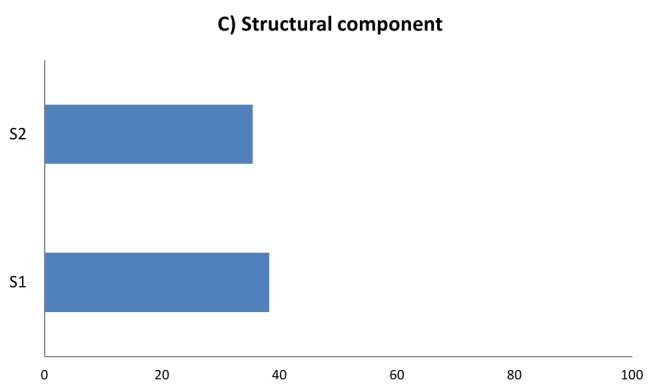
S1: Previous events affecting the safety of hospital buildings S2: Safety of structural systems and materials used in building

In regard with the non-structural elements, the average safety score ranged from 31.1 to 41.3 for the “office and storeroom furnishings and equipment” and “electrical system”, respectively (table 5). Figure 1-B illustrates this. The average safety score was 36.0 out of 100 (±13.9 SD) for all elements of non-structural component.

**Table 5 – Average score of non-structural safety for disasters in 224 Iran’s hospitals, 2012:** *MOH&ME: Ministry of Health & Medical Education; †SWO: Social Welfare Organization; NS1: Electrical system; NS2: Telecommunications system; NS3: Water supply; NS4: Fuel storage; NS5: Medical gases; NS6: Heating, ventilation, and air-conditioning (HVAC) systems in critical areas; NS7: Office and storeroom furnishings and equipment (fixed and movable); NS8: Medical and laboratory equipment and supplies used for diagnosis and treatment; NS9: Architectural elements

	**NS1**	**NS2**	**NS3**	**NS4**	**NS5**	**NS6**	**NS7**	**NS8**	**NS9**	**All elements**
**Hospital affiliation **										
MOH&ME*	40.5	34.7	34.5	41.4	32.1	33.6	31.3	32.6	37.2	35.5
Private	45.5	40.1	42.9	43.3	37.1	40.1	35.5	39.6	41.0	40.7
SWO†	40.6	35.4	33.6	39.5	36.1	34.0	33.3	31.6	38.8	36.2
Military	45.8	39.0	38.6	38.3	23.8	26.6	28.8	31.6	37.4	34.9
Charity	40.2	26.9	24.4	38.8	20.6	22.2	11.1	21.3	25.3	25.8
*P value*	*0.70*	*0.49*	*0.31*	*0.98*	*0.32*	*0.36*	*0.33*	*0.12*	*0.63*	*0.31*
**Hospital function**										
General	41.0	35.8	34.9	40.6	32.5	34.1	31.9	33.0	37.7	35.9
Specialized	42.9	33.7	36.9	44.3	33.4	34.0	30.5	33.4	37.2	36.3
*P value*	*0.47*	*0.45*	*0.55*	*0.36*	*0.78*	*0.99*	*0.66*	*0.88*	*0.86*	*0.89*
**Hospital size**										
≤100 beds	41.0	35.2	34.5	40.0	31.8	33.0	31.1	32.7	37.0	35.4
>100 beds	42.7	36.4	38.6	47.0	36.6	38.5	34.1	34.9	40.0	38.6
*P value*	0.55	0.66	0.24	0.07	0.12	0.10	0.36	0.38	0.33	0.16
**All hospitals**	41.3	35.4	35.3	41.4	32.7	34.1	31.1	33.1	37.6	36.0

As presented in table 6, the average safety score of various elements of structural safety ranged from 35.4 to 38.4 for the “safety of structural systems and materials used in building” and “previous events affecting the safety of hospital buildings”, respectively. Figure 1-C visualizes this. The average safety score out of 100 was 36.0 (±19.0 SD) for all elements of structural component.

**Table 6 – Average score of structural safety for disasters in 224 Iran’s hospitals, 2012:** *MOH&ME: Ministry of Health & Medical Education; †SWO: Social Welfare Organization; S1: Previous events affecting the safety of hospital buildings; S2: Safety of structural systems and materials used in building

	**S1**	**S2**	**All elements**
**Hospital affiliation **			
MOH&ME*	38.0	34.5	35.3
Private	40.0	40.8	40.6
SWO†	38.3	37.0	37.3
Military	42.2	36.0	37.4
Charity	33.3	25.5	27.3
*P value*	*0.97*	*0.57*	*0.66*
**Hospital function**			
General	39.2	35.5	36.4
Specialized	34.3	34.7	34.6
*P value*	*0.17*	*0.81*	*0.58*
**Hospital size**			
≤100 beds	38.5	34.9	35.7
>100 beds	37.2	37.9	37.4
*P value*	*0.71*	*0.44*	*0.59*
**All hospitals**	38.2	35.4	36.0

Neither the safety classes nor the scores of safety components were significantly associated with types of hospitals in terms of affiliation, function and size (P>0.05). Accordingly, we did not observed a significant difference for the scores of safety elements among various types of the hospitals (P>0.05). The only exceptions were higher score related to two elements of functional capacity in large hospitals (>100 beds), i.e., “organization of hospital disaster committee (HDC) and emergency operations center (EOC)” and “contingency plans for medical treatment in disasters” (P<0.05).

The following 10 hazards were reported by the hospitals as the hazards with highest probability of occurrence and impact: Earthquake 68%, dust storm 62%, extreme temperature 58%, hospital overload 49%, power outage 49%, water cut 48%, fire 46%, torrential rains 46%, storms 44% and landslides 44%.

## Discussion

Our study showed that the overall disaster safety status of the Iran’s hospitals was 33 out of 100. More than half of the hospitals were found as low safe and the rest were moderately safe. Accordingly, we did not observe any hospital in the high safe category.

Hospitals safety assessment using the HSI shows significant variation by region. The assessments on 45 hospitals in Caribbean countries revealed that only 2% of the hospitals were completely safe, 80% were moderately safe and 18% were low safe 9. While the assessments on 66 hospitals in Maldova classified 24.6% of the hospitals as high safe, 67.2% as moderate safe and only 8.2% as low safe 10. The results derived from the HSI can be considered as indicators for measurement of the health system resilience for disasters.

To our best knowledge, this is the first study in Iran that reports the disaster safety status of a large number of hospitals, i.e. about 1/4 of the country hospitals. While the response rate was not high, it was expected for the first year of the program. To ensure the hospitals participation, the disaster safety status must be included in licensing and accreditation of the hospitals. This strategy will be a self-motive for the hospitals to follow the MOH&ME policy.

Low response rate potentially raises the concern about occurrence of a selection bias. From experience and to our best knowledge about the hospitals disaster mitigation and preparedness programs in Iran, we do not expect a meaningful difference between responding and non-responding hospitals. However, a complete survey of all hospitals is our goal.

While our assessment using the FHSI, does not replace detailed vulnerability and preparedness studies, it is able to provide an overview of hospitals safety status for disasters in Iran. This information can be used by decision makers as the basis for policy making, planning and resource allocation. Repeating the assessments on an annual basis will help the health system to evaluate the effectiveness of intervention programs and its trend over time.

As it is emphasized by WHO, HSI is a low-cost and rapid diagnostic tool 7. We relied on these characteristics because of the feasibility concerns associated with large number of hospitals in the country and cost of alternative detailed assessments. While we do believe that the self-assessment strategy was the limitation of our survey, it was the only way that was possible at the time of the program initiation. Furthermore, the MOH&ME believes that the self-assessment compared to external assessment will be a sustainable strategy over time because of limited resources.

To address the aforementioned limitation, in October 2013, the MOH&ME in collaboration with WHO held the first workshop to train a group of professional assessors. The certified assessors were from nine disaster management regions of the country and are mandated to provide the hospitals with technical guidance in their own regions. In addition, they have been trained to evaluate the validity of the self-assessment strategy through a stratified random sampling of the hospitals. This validity study will help the health system to determine the sources of errors and quantifies the magnitude of potential bias that may be caused by self-assessment.

The self-assessment approach, however, is useful for training of the hospitals disaster committees. In the FHSI like its original version, each item is followed by its own specific guide that presents the standards of the safety. This helps the hospitals to simultaneously learn what the expected standards are.

We did not observe any difference between various types of hospitals regarding their safety status in terms of functional capacity, non-structural and structural components. The only exceptions were better scores in large hospitals versus small hospitals in terms of “organization of HDC and EOC” and “contingency plans for medical treatment in disasters”. So, it is clear that all hospitals are in need of safety interventions. But the limited resources require that Iran’s health system prioritize its investments over time. Classification of the hospitals safety to low, average and high classes helps the MOH&ME to prioritize the hospital targets. To do this, another key factor must be taken into account, i.e., the role of a hospital in the emergency plan of a defined area. For instance, the MOH&ME and SWO affiliated hospitals are expected to be more involved in response to external hazards. So, they must be considered as the first priority for intervention. In another word, the MOH&ME and SWO affiliated hospital with low and average safety levels are recommended for first phase of intervention programs.

In 2010, MOH&ME performed a structural safety assessment on public hospitals. The focus of the assessment was on earthquake resistance using a rapid visual screening (RVS) method. The results showed that the structure of about 70% of the assessed hospitals were not safe for an earthquake. Accordingly, a 10-year plan was defined to enhance the structural safety of the hospitals, although its budget has not been secured yet. While the structural resistance are of high importance during a disaster 4 ^,^ 5 ^,^ 7 ^,^ 11, the retrofitting process requires considerable amount of budget and technical capacity. So, while we need to work on fund raising and capacity building for the structural safety, at the same time we must focus on other components that require fewer budgets. Studies have showed that many hospitals have failed during a disaster because inappropriate emergency plan and/or non-structural damage 12 ^-^ 15. Compared to structural component, fortunately these components need fewer budgets to be addressed. This approach does not prevent the hospital failure in large disasters such as a severe earthquake, but it works well in small and moderate scale disasters.

In addition to the existing hospitals, we are also concerned about safety of over 100 hospitals that are under construction in Iran by MOH&ME, SWO, Ministry of Road and Urban Construction and private sector. Since, we even recently witnessed destruction of the newly opened hospitals such as the case of 2012 earthquake in East Azarbaijan province, there is no doubt that Iran’s health system should enhance its supervision on appropriate implementation of the safety codes in construction of new hospitals. Furthermore, enhancement of technical capacities in all aforementioned partners is recommended.

Protecting of health facilities from disasters has been considered in the road map of Iran’s health system for disaster risk management 16. To achieve this, a multi-disciplinary collaboration and a commitment from high level of authorities are required. In this line, we suggest establishment of a national committee for hospital safety in disasters. This committee should include all key stakeholders including MOH&ME, SWO, military, Islamic Parliament, Ministry of Road and Urban Construction and private sector. Since the MOH&ME is in charge of hospitals licensing and accreditation, its relevant centers/offices are also recommended to represent in this committee. These centers/offices include Hospitals Accreditation and Supervision Center, Hospital Management Center, Physical Resources and Construction Office and Disaster & Emergency Management Center.

In summary, to enhance the hospitals safety for disaster in Iran, we wish to recommend: 1) establishment of a national committee for hospital safety in disasters including MOH&ME, SWO, military, Islamic Parliament, Ministry of Road and Urban Construction and private sector; 2) supervision on implementation of the safety standards in construction of new hospitals; 3) enhancement of functional readiness and safety of non-structural components while structural retrofitting of the existing hospitals is being taken into consideration, whenever is cost-effective; and 4) FHSI should be considered in licensing and accreditation of the hospitals. This would encourage the hospitals to invest on disaster mitigation and preparedness.

## Competing Interests

The authors have declared that no competing interests exist.
